# The Risk of Undeclared Allergens on Food Labels for Pediatric Patients in the European Union

**DOI:** 10.3390/nu14081571

**Published:** 2022-04-10

**Authors:** Montserrat Martínez-Pineda, Cristina Yagüe-Ruiz

**Affiliations:** Faculty of Health and Sports Science, University of Zaragoza, 22002 Huesca, Spain; cyague@unizar.es

**Keywords:** undeclared allergens, pediatric, food allergies, risk, RASFF

## Abstract

The dietary avoidance of allergens has been widely recognized as the key intervention in the management of food allergies, but the presence of undeclared allergens makes compliance difficult. The aim of this study was to analyze the presence of undeclared allergens in food labeling through RASFF notifications in the European Union, focusing on those allergens that frequently affect the pediatric population and the implicated products, so as to provide useful information for its risk evaluation and the development of educational materials for patients. The results showed milk (20.5%), gluten (14.8%), and nuts (10.9%) to be the pediatric allergens with higher presences. In 80% of the notifications concerning milk and milk derivatives, the specific compound present (lactose or lactoprotein) was not identified. They were mainly present in cereal and bakery products, prepared dishes and snacks, and cacao and confectionery products, all of which are frequently consumed by the pediatric population. The large quantity (7.6%) of undeclared allergens in “free-from-allergen” products was also remarkable, especially in regard to the supposedly not-present allergens. Undeclared allergens in food products pose an evident risk for allergic patients and knowledge of them should take a relevant role in a patient’s nutritional education. It is also necessary to raise awareness among manufacturers and safety authorities.

## 1. Introduction

The prevalence of allergic diseases worldwide is rising dramatically in both developed and developing countries. These diseases include asthma; rhinitis; anaphylaxis; drug, food, and insect allergies; eczema; urticaria (hives); and angioedema. This increase is especially problematic in children, who are bearing the greatest burden of the rising trend that has occurred over recent years [1]. Among allergic diseases, it is generally accepted that food allergies (FAs) affect approximately 2.5% of the general population, but the spread of prevalence data is wide, ranging from 1% to 19%, depending on patient age, diagnosis criteria, geographic area, etc. [2] In Europe, the estimated prevalence of FAs ranged from 1.0% to 5.6% in school-age children, while food sensitization (FS) ranged from 11.0% to 28.7%. Both primary and cross-reactive FS and FA occurred frequently at this age, according to data provided by Lyons et al. (2018) [3]

In children, the foods that frequently trigger allergic reactions include eggs, cow’s milk, peanuts, tree nuts, soy, and wheat. Although some allergies typically resolve during childhood, allergies to peanuts and tree nuts, as well as those to fish and shellfish, remain into adulthood [4]. In children and adolescents <18 years of age, a systematic review that included 42 studies published in Europe between 2000 and 2012 reported a higher prevalence of food-challenge-defined allergies to cow’s milk, 0.6% (0.5–0.8), followed by tree nuts, 0.5% (0.08–0.8), soy, 0.3% (0.1–0.4), eggs, 0.2% (0.2–0.3), peanuts, 0.2% (0.2–0.3), wheat, 0.1% (0.01–0.2), fish, 0.1% (0.02–0.2), and shellfish, 0.1% (0.06–0.3) although these percentages tended to be higher when allergy data were self-reported [5].

The clinical management of food allergies includes short-term interventions to manage acute reactions and long-term strategies to minimize the risk of further reactions. The strict dietary avoidance of allergens has been widely recognized as the key intervention in the management of FAs, resulting in the complete or almost complete resolution of symptoms [6]. In order to properly adhere to recommended elimination diets, patients and families should be instructed to pay careful attention to ingredient lists and food labels.

European legislation (EU Regulation No. 1169/2011) requires that information on the presence of allergens in foods is always provided to consumers, including on non-prepackaged foods. This regulation requires the indication of the presence of the 14 substances or products causing allergies or intolerances (hereinafter referred to as “allergens”) shown in Table 1 when incorporated into food as ingredients [7]. This list was established on the basis of the scientific opinions adopted by the European Food Safety Authority (EFSA) [8]. 

With regard to prepackaged foods, allergen information must appear in the list of ingredients, with clear references to the names of the substances or products given in Table 1. In addition, it should be highlighted by a typographical composition that clearly differentiates it from the rest of the list of ingredients (e.g., by typeface, style, or background color). In the absence of a list of ingredients, the word “contains” must be included, followed by the substance or product as listed in Table 1. An indication shall not be required in cases where the name of the food clearly refers to the substance or product causing allergies or intolerances.

With respect to non-prepackaged foods, the member states are allowed to adopt national measures concerning the means through which information on allergens on these foods is to be made available [7]. For example, in Spain, this is regulated by Royal Decree 126/2015 of 27 February 2015, which approves the general rule of food information on foodstuffs presented as unpackaged for sale to the final consumers and mass caterers, those packaged at the point of sale at the request of the purchaser, and those packaged by retail trade operators [9].

The food industry produces foods free of certain ingredients for consumers with food allergies or intolerances. In the European Union, there is legislation that regulates the requirements for the provision of information to consumers in the absence or reduced presence of gluten in food (EU Regulation No. 828/2014) [10]. This information should help gluten-intolerant people to identify and choose a varied diet when eating inside or outside their homes. There is also legislation regulating statements relating to the presence or absence of lactose in infant formula and follow-on formula (EU Regulation No. 2016/127), which can provide useful information to parents and caregivers [11]. However, there are still no harmonized rules at the EU level on labeling and composition indicating the absence or reduced presence of lactose in other foods. Given the importance of these claims for lactose-intolerant people, some member states have adopted non-binding guidelines. For example, the claims “lactose-free” and “low lactose” are used on foodstuffs for ordinary consumption marketed in Spain when the foodstuff contains less than 0.01% and 1% lactose, respectively [12].

The mandatory indication of allergenic compounds on labels is a very useful tool for patients to avoid consuming foods that contain allergens in their formulations. However, despite being mandatory, food mislabeling is on the rise and does not always adequately contain information about the allergens present [13], which implies a risk for allergic patients. Since the presence of undeclared allergens in food labeling has been considered to be a public health risk for a certain population, they have been included in the RASFF system [14]. The RASFF system database was created by the European Commission to keep the latest information on food recalls and public health warnings in all European Union (EU) countries, as well as Norway, Liechtenstein, Iceland, and Switzerland.

As a direct risk for allergic patients, the aim of the present study was to analyze the presence of undeclared allergens in food labeling through the European food-allergen-related notifications published on the RASFF portal from 2018 to 2021. Likewise, the analysis focused particularly on the undeclared presence of allergens that more frequently cause allergic reactions in the pediatric population, as well as on the food products that contain them, as useful information for patients’ potential risk evaluation of commercial food products and as a relevant tool for the development of educational materials for families.

## 2. Materials and Methods

The data were obtained directly from the EU RASFF open data portal in .xlsx format [15]. The following items were available for each notification: date of notification; notifying country; origin country; type of product (food, food contact material, or feed); product category; product involved; hazard category; substance/finding; full hazard; subject; notification type; type of control; risk decision; distribution status; action taken after notification; and result (if available). From this online open database, notifications for the period between 1 January 2018 and 31 December 2021 were extracted under the hazard category filter “allergens” and type of product “food”.

The following data were studied: date of notification; notification type; notifying country; origin country; type of control; main allergen hazard; product category; action taken after notification; and food product involved. 

### 2.1. Main Allergen Hazard

This item refers to the allergen(s) involved in the notification. RASFF system data provide this information, grouping allergens according to the mandatory declaration allergens in prepackaged and non-prepackaged food products listed in Table 1. In some cases, the database provides specific information about the allergen, e.g., “wheat” or “oats” instead of “cereal containing gluten”; “lactose” or “lactoprotein” instead of “milk”; or a particular tree nut instead of “nuts”. In the results section, these particular allergens were included and treated according to the mandatory declaration allergen groups to which they belong. Regarding crustaceans and mollusk allergens, when it was convenient for clarifying explanations, they were grouped and listed as “seafood allergen”.

Furthermore, for all EU mandatory declaration allergens, the study paid particular attention to the analysis of those allergens of the greatest interest for the allergic pediatric population due to their higher prevalence.

### 2.2. Food Category 

In addition to the main allergen hazard, the database gave a detailed description of the product involved in the notification (e.g., wafer rolls with cream). The product was also classified within one of the 27 food categories established by the RASFF system: alcoholic beverages; bivalve mollusks and products thereof; cephalopods and products thereof; cereals and bakery products; cocoa and cocoa preparations, coffee, and tea; confectionery; crustaceans and products thereof; dietetic foods, food supplements, and fortified foods; eggs and egg products; fats and oils; fish and fish products; food additives and flavorings; fruits and vegetables; gastropods; herbs and spices; honey and royal jelly; ices and desserts; meat and meat products (other than poultry); milk and milk products; natural mineral water; non-alcoholic beverages; nuts, nut products, and seeds; other food products/mixed; poultry meat and poultry-meat products; prepared dishes and snacks; soups, broths, sauces, and condiments; and wine.

### 2.3. Statistical Analysis

A descriptive statistical analysis of the data (proportions) was carried out for each of the items studied: notification type, action taken after notification, main allergen hazard, product category, and food product involved. Descriptive statistical analyses were performed with Microsoft Excel 2016^®^ (Microsoft Corporation, Redmond, WA, USA).

A one-way ANOVA, followed by a Tukey post-hoc test, was applied to determine the statistical differences in the total number of undeclared allergen notifications among pediatric-relevant allergens (milk, gluten, nuts, soybean, egg, peanut, fish, crustaceans, and mollusks) realized in the period between 1 January 2018 and 31 December 2021. Values of *p* < 0.05 were considered significant. A two-way analysis of variance (ANOVA), followed by a Tukey post-hoc test, comparison test was performed to determine statistical differences in the total number of undeclared allergen notifications between years and allergens, as well as differences in the main pediatric allergens per food category. The same threshold for statistical significance (*p* < 0.05) was considered. These data were analyzed using GraphPad Prism version 6.01 (GraphPad Software, Inc., San Diego, CA, USA).

## 3. Results

Between 1 January 2018 and 31 December 2021, a total of 844 food-allergen-related notifications were made by the RASFF system, the year 2019 being the one with the highest number of notifications, *n* = 241, while the years 2018, 2020, and 2021 had *n* = 207, *n* = 197, and *n* = 199, respectively. It should be remarked that, in the period studied, 79.7% of the notifications were classified as an “alert”, which implies that the food presents a serious risk on the market, requiring a rapid action generally aimed at withdrawing the product from the market. However, “information for attention and for follow-up” notifications represented only 17.4%. These types of notifications concern information that does not require a rapid action because the food product containing the undeclared allergen is still not on the market at the time of the report, or the risk is mostly considered low.

It is also relevant that, of the 844 allergen notifications reviewed, 16.6% corresponded to foods that contained two or more undeclared allergens, and could therefore potentially affect people with different allergies. Table 2 shows the proportion of each of them. It should be noted that several of these notifications revealed the presence of allergens that frequently affect children in products that easily could be consumed by them, for example, a 2018 notification that alerted about milk, soy, and wheat (in addition to mustard and celery) in organic beetroot soup.

It can be observed that, from all those notifications, the main countries that emitted notifications about allergen hazards were Belgium, the Netherlands, and the United Kingdom. However, it should be remarked that 2021 data did not report notifications from the United Kingdom since it was no longer part of the EU as of January 2021. Regarding the countries of origin of the products concerned, it was observed that the main provenance was EU countries (77.6%), while 22.4% of notifications implied a non-EU/EEA country as the origin of the food.

From all of these notifications, it can be seen that the detection of the undeclared allergen in the food product came from a company’s own check (51.1%), followed by official controls on the market (32.8%), and consumer complaints (9.6%). Notifications due to food poisoning represented 2.1% of the total notifications. These percentages remained very similar over the years studied.

**Figure 1:**
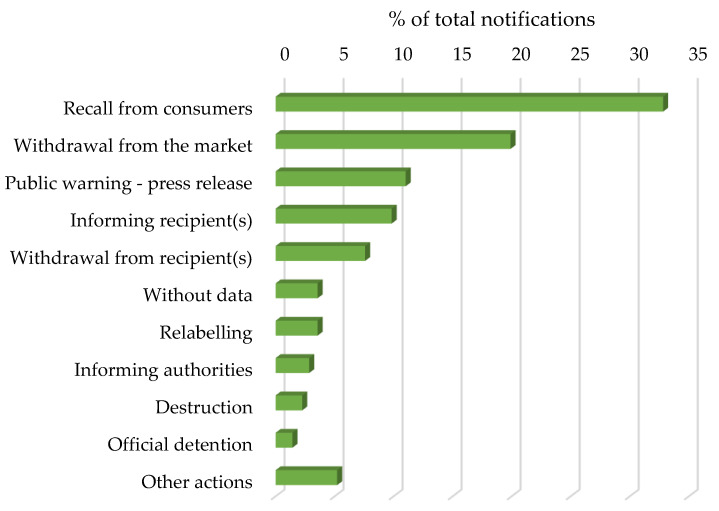
Percentage of actions taken as responses to notifications received between 1 January 2018 and 31 December 2021.

In terms of responses to the notifications, the most frequently taken actions were the foods being recalled from the consumers (38.2%) and withdrawn from the market (19.9%) (Figure 1). All of these results are very relevant since they imply that the food products that contained the undeclared allergens were already on the market and could have already been consumed by an allergic person.

### 3.1. Main Allergen Hazard

**Figure 2:**
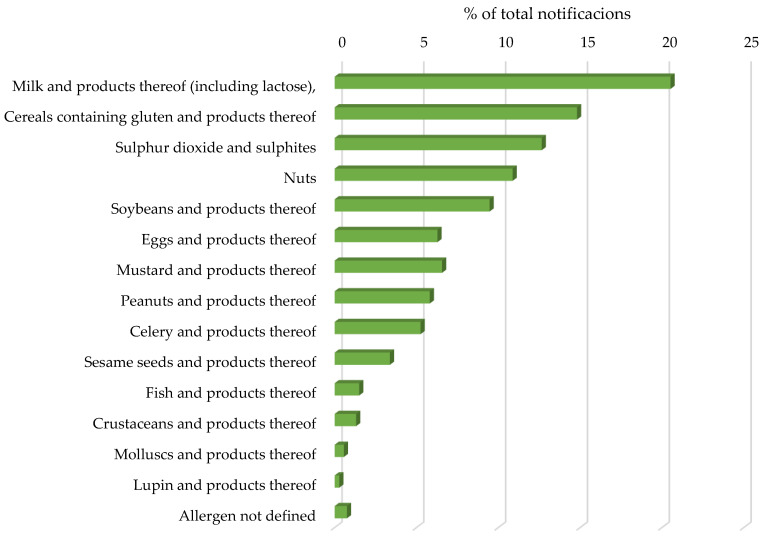
Percentage of total notifications per mandatory declaration allergen in the EU between 1 January 2018 and 31 December 2021.

According to the data, during the period studied, the main allergens about which notified were emitted were milk and products thereof, followed by cereals containing gluten and products thereof, sulfur dioxide and sulfites, and nuts (Figure 2), two of them (milk and nuts) being allergies with a higher prevalence in the pediatric population. 

Apart from milk and its derivatives and nuts, other potential risk allergens in the pediatric population due to their high allergy prevalence, such as gluten, soy, eggs, or peanuts, were also involved in 36.4% of the notifications. On the other hand, it was found that the allergens that were the least frequently mentioned in the notifications were fish and seafood (crustaceans and mollusks), representing 1.5% and 1.9% of the notifications, respectively. 

**Figure 3:**
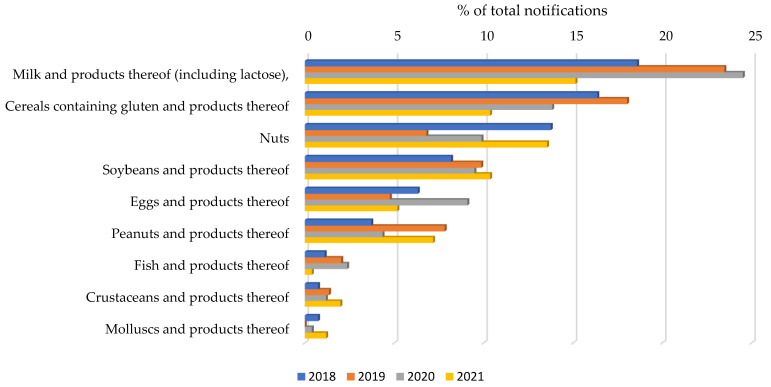
Percentage of total notifications of the main allergens affecting the pediatric population per year.

**Figure 4:**
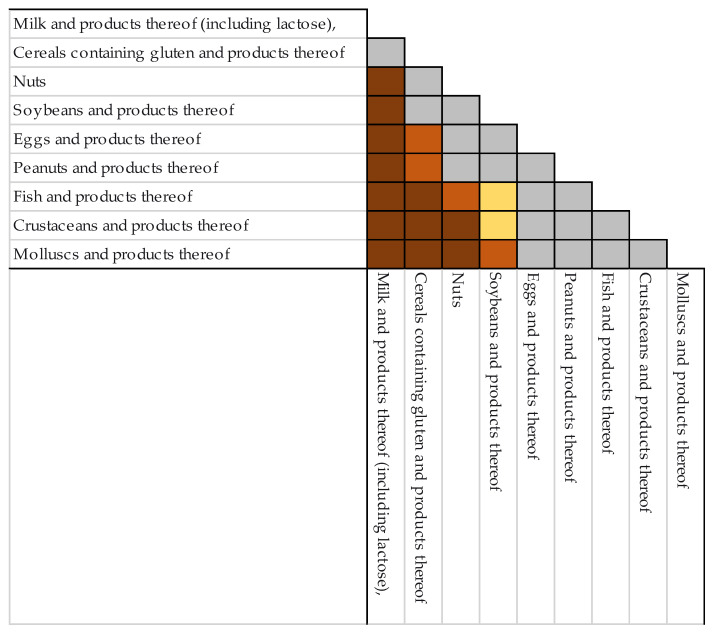
Statistical differences among the undeclared, main pediatric allergens in notifications emitted between 1 January 2018 and 31 December 2021. In grey- no significant differences; in yellow- significant differences with *p* < 0.01; in orange - significant differences with *p* < 0.005; and in brown-significant differences with *p* < 0.001.

The evolution of notifications-per-year of the main allergens that affect children and adolescents is shown in Figure 3. Globally, during the period studied, there were observed significant statistical differences between these allergen notifications (Figure 4); however, it should be remarked that, during the period of study, “milk and products thereof” remained the allergen that triggered the most notifications. Regarding differences in each allergen per year, the number of "undeclared milk notifications was significantly higher in 2019 and 2020, *p* < 0.01 and *p* < 0.05, respectively, compared to those of the year 2021, and regarding gluten notifications, the number was significantly higher (*p* < 0.05) in 2019 than in 2021. For the rest of the allergens, no significant differences were found over the four years.

**Figure 5:**
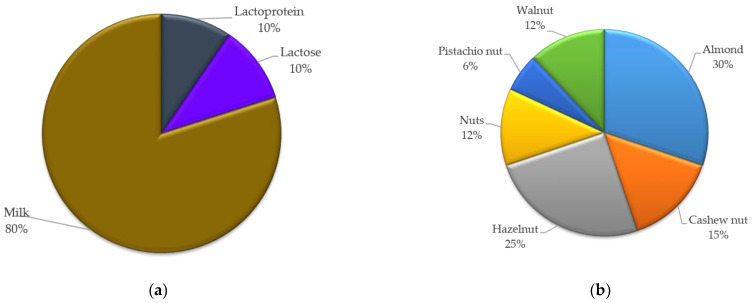
Percentage of notifications related to undeclared milk and products thereof as well as nuts that specified the concrete compounds present in food products: (**a**) percentage of notifications that detailed milk-related allergens; (**b**) percentage of notifications that detailed nut-related allergens.

Figure 5 shows specific allergen notifications related to milk and nuts. In the case of milk and products thereof, only 20% of the related notifications specified the concrete compound present (lactose or lactoprotein), while the rest of the notifications only indicated the presence of milk. Conversely, 88% of the nut-related notifications specified the nuts involved; most of the notifications referred to the undeclared presence of almonds and hazelnuts. This information is relevant since a person could be lactose-intolerant but not necessarily allergic to a lactoprotein. If the response to a notification is a public warning (e.g., a press release), the use of the general term “milk” would not provide enough information to consumers.

In regard to almost all of the allergens that frequently affect the pediatric population, it was detected that the RASFF notifications were mainly due to labeling errors rather than the presence of the allergen or its traces due to possible cross-contamination. Among the labeling errors detected, it was also observed that some products that included information in several languages did not inform about the presence of allergens in one language but did in another. 

The RASFF system included, in some cases, but not systematically, the quantity of the undeclared allergen in the product. With the available data, regarding milk and products thereof, it could be observed that the amount of undeclared milk (or milk ingredients) ranged between 0.35 mg/kg to 150 g/kg. The presence of lactoprotein in related notifications varied from 0.5 to 2500 mg/kg, and for undeclared lactose, quantities between 0.36 mg/kg and 15.6 g/kg were detected. In the case of undeclared peanuts, amounts between 1.2 mg/kg and 86.3 g/kg were found.

It should also be remarked that, for the undeclared gluten notifications, 23.2% of them were for gluten-free products due specifically to their gluten content being too high. According to EU Commission Implementing Regulation No. 828/2014 of 30 July 2014 on the requirements for the provision of information to consumers on the absence or reduced presence of gluten in food, the statement “gluten-free” may only be made where the food as sold to the final consumer contains no more than 20 mg/kg of gluten [10]. In all of these notifications, the gluten content exceeded that limit, being in some cases close to 1000 mg/kg. This quantity was as high as 1600 mg/kg in other products that were not labeled as “gluten-free,” but which contained undeclared gluten.

### 3.2. Food Categories and Related Allergens

**Figure 6:**
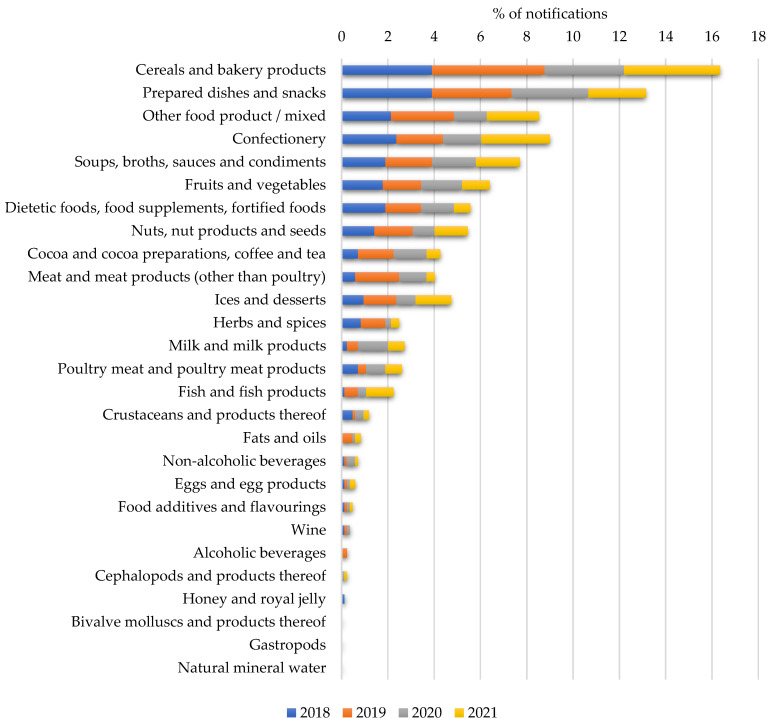
Percentage of notifications according to food categories per year.

The notification percentage of each food category per year is shown in Figure 6. The results show that the main categories subject to notifications related to undeclared allergens were those of cereals and bakery products (16.3%), prepared dishes and snacks (13.1%), and confectionery (9.0%), followed by others that were classified as mixed products (e.g., frozen veggie burgers) (8.5%) and soups, broths, and sauces/condiments (7.7%). In general, those categories that include food products with a higher degree of processing and number of ingredients proved to be the ones that collected the highest number of notifications. The specific foods included in each category, according to what is stated in the RASFF database, are presented in the Supplementary Material (Table S1).

Fruits and vegetables ranked sixth among the reported food categories. It should be clarified that the products involved were usually fruits and vegetables with different degrees of handling (sliced, grated, powder, pulp, purée, spread, and dried) or processing (dried, pickled, canned, and preserved), or vegetable mixtures.

**Figure 7:**
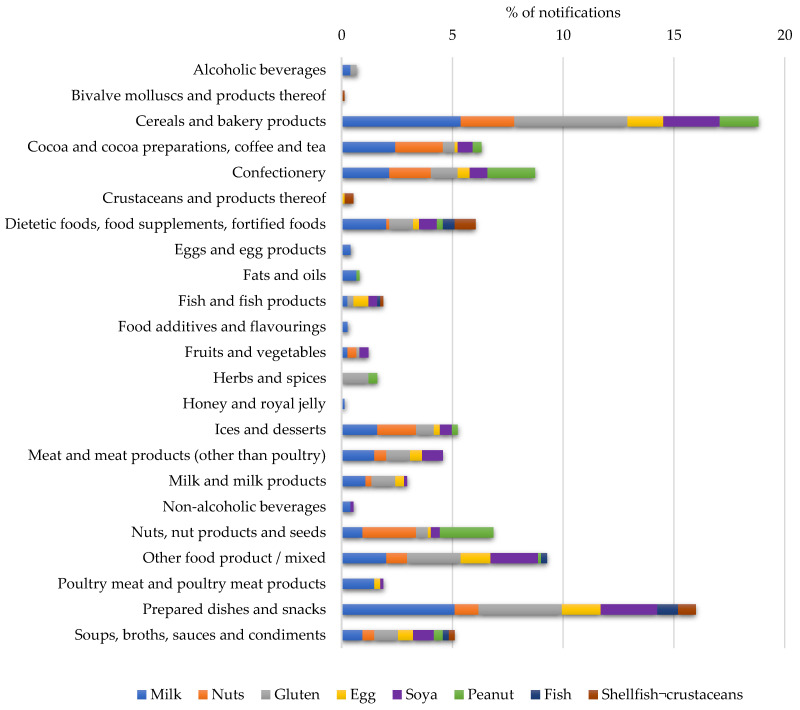
Relationship between the main allergens affecting the pediatric population and their presence within each food category.

Figure 7 shows the relationship between the main allergens affecting the pediatric population and their presence within each food category. The results revealed that soups, broths, sauces, and condiments, in addition to dietetic foods, food supplements, and fortified foods were the categories which included all common pediatric allergens (milk, nuts, gluten, egg, soy, peanut, fish, and seafood allergens). These categories were followed by prepared dishes and snacks as well as other mixed products which contained all allergens except peanut and seafood allergens, respectively. The types of food products included in the other/mixed products category are shown in Table A1 (Appendix A).

The presence of undeclared milk was significantly higher (*p* < 0.005) in cereal and bakery products as well as in prepared dishes and snacks. Similar behavior was detected for gluten, with its undeclared presence also being significantly higher (*p* < 0.001 and *p* < 0.05, respectively) in these two food categories compared with the remainder oof the categories. Regarding these two allergens, no significant differences in the number of notifications were observed among any of the other categories.

For the rest of the allergens, no significant statistical differences between the categories were observed. However, undeclared nuts were found predominantly in the cereal and bakery products category, nuts, nut products, and seeds, as well as cocoa and cocoa preparations. It should be noted that products included in these categories, especially the bakery and cocoa ones, are frequently ingested by infants.

For their parts, soy and eggs were mainly present in cereal and bakery products, as well as in prepared dishes and snacks. These results were expected since these foods are widely used as ingredients in both categories due to their technological functionalities, for example, as emulsifiers. Moreover, as both ingredients are widely used, the risk of cross-contamination within the industry is increased.

The presence of undeclared peanuts was higher in nuts, nut products, and seeds, as well as in the confectionary category. This can be easily explained due to cross-contamination because, although peanuts are legumes, their use and consumption are frequently linked with nut products (e.g., roasted, mixed nuts). Fish- and seafood-allergen-related notifications were mainly caused by dietetic foods and food supplements, but also by prepared dishes and snacks.

It should be noted that the cereal and bakery products category, in addition to being the one that caused the most notifications, warned of the undeclared presence of all of the most frequent allergens in children, except fish and seafood allergens. Most of the notifications in this food category were due to the presence of milk and products thereof, as well as cereals containing gluten.

It is especially noteworthy that 5.9% (*n* = 50) of the notifications were caused by gluten-free products. From them, 78% were due to the presence of gluten, while the remaining 22% were due to other allergens, such as lactose, milk, soy, or nuts, such as hazelnuts. The gluten-free products mainly involved were bars, biscuits, breads, cakes, chips, cookies, crisps, flours, granolas, muffins, pastas, noodles, nuggets, pizza toppings, ice creams, and desserts, among others. It should also be noted that many of them were also organic products. Additionally, other specially designed products for allergic or intolerant patients presented undeclared allergens, as can be observed in Table 3. These results are relevant since, in the case of allergic patients, their ingestion could directly affect their health status.

In this context, products especially designed for covering alternative dietary patterns, such as those of vegetarians and vegans, which included statements such as “veggie”, “vegetarian”, or “vegan”, represented 4.4% (*n* = 37) of the total notifications. Of them, 64.9% referred to undeclared allergens of animal origin (56.8% milk and 16.2% egg), while 35.1% were non-animal-related allergens (mustard, nuts, soy, celery, gluten, and peanuts).

Food products specifically intended for babies were also the subject of some notifications. This was the case for infant starter milk with the presence of fish allergens; baby cereal porridge with undeclared milk, lactose, and soya; and undeclared gluten in organic, gluten-free baby food.

## 4. Discussion

As there is no curative treatment for food allergies, and allergen avoidance is the mainstay of management, the presence of undeclared allergens in food implies a significantly dangerous risk for allergic patients. The avoidance of food allergens is onerous for patients and families and often fails, with 10% of patients on average experiencing at least one allergic reaction per year [16]. In their study, Fleischer et al. established that 87.4% of allergic reactions to foods in preschool-aged children were mainly caused by accidental exposure. Among causes of accidental reactions, unintentional ingestion (e.g., purely accidental due to forgetfulness, reduced supervision, not checking a product, etc.), label-reading errors, cross-contamination, errors in preparation, and manufacturer’s labeling errors were found. The severity grade of these allergic reactions varied considerably, both among individuals and the allergens involved. Of all of the allergic reactions registered by the authors, 70.1% involved mild symptoms (skin, and/or oral symptoms, and/or upper-respiratory symptoms, but not all three organ systems); 18.4% involved moderate symptoms (skin, oral, upper-respiratory, or gastrointestinal symptoms); and 11.4% involved severe reactions (lower-respiratory symptoms; cardiovascular symptoms; or a combination of skin, oral, upper-respiratory, and gastrointestinal symptoms). These results reveal the impact of individual sensitivity to a particular allergen [17].

Although food-related anaphylaxis is relatively common, and all allergens are likely to cause severe reactions, fatalities remain rare, with a reported range of approximately 0.03 to 0.3 deaths per million people per year in the general population, and are very rare in infants and young children [18]. However, the vast majority of fatal allergic reactions were due to peanuts, tree nuts, seafood, and cow’s milk [19]. Moreover, the consumption of non-prepackaged foods, served in catering establishments, self-service stores, bakeries, restaurants, etc., is frequently involved. This could probably be associated with a lack of direct allergen information on these kinds of products and the high risk of cross-contamination [20].

Despite the efforts made by the European Union to increase controls and consumers’ information through labeling, including in community legislation regarding the mandatory declaration of allergens in food products, the results of the present study show that the risk due to the presence of undeclared allergens continues to be a problem for these patients. Previous studies have already highlighted that the presence of undeclared allergens was one of the main causes of food safety incidents/recalls [21]. An analysis of global recalls from previous years (from 2008 to 2018) also placed milk as the most frequently undeclared allergen, along with multiple allergens and gluten [22]. According to our results, this trend has persisted over time. However, contrary to what was observed in our results, nuts were not listed among the top allergens with the highest incidence in analyses of previous years and of non-EU countries [23,24]. Data from different countries and continents (RASFF vs. CDC, New Zealand, Australia, etc.), as well as the allergen-monitoring increase throughout the world in recent years, associated with the development of specific regulatory legislation, could explain the observed differences.

Previous studies also established cereal and bakery products as the most common food categories associated with the undeclared presence of milk, followed by confectionery [22]. However, this has changed over the four years of the study period since, from our results, it can be observed that milk allergens were more common in the recalls of prepared dishes than in those of confectionery. In any case, prepared dishes have already been identified by previous analyses as frequent causes of recalls [24]. The obtained results are worrying since both categories, derived from cereals and prepared dishes, are likely to be frequently consumed by children and adolescents, and they lead the list of products with the most prevalent allergens for this population.

FA patients often consume food products that self-report to be allergen-free (e.g., dairy-free) on their labels, trusting in their safety. However, the results exposed a concerning presence of undeclared allergens in a wide variety of those foods. This fact was especially striking in prepared meals and bakery products classified as “gluten-free”. The evidence regarding the threshold limit of gluten concentration in food is also unclear. The consumption of about 200 mg gluten per day is clearly associated with the development of intestinal mucosal abnormalities after only 4 weeks in patients with celiac disease. However, it has been demonstrated that individual sensitivity to gluten varies among people with celiac disease, and the daily-intake limit should lie between 10 and 100 mg [25]. The length of exposure to gluten is also a determinant factor. For example, some authors have found that the ingestion of 10 or 50 mg gluten per day was associated with the worsening of the villous height/crypt depth ratio in most patients after 3 months [26]. In any case, under these premises, the high gluten amounts reported in some notifications (from 20 to more than 1600 mg/kg) highlight the obvious risk for some people with celiac disease and gluten intolerance, as the maximum limit ingestion must not exceed the 10 mg per day to avoid detrimental effects, such as significant histological abnormalities in some patients. According to European legislation, the statement “gluten-free” may only be made when the food as sold to the final consumer contains no more than 20 mg/kg of gluten, and the statement “very low gluten” may only be made where the food, consisting of or containing one or more ingredients made from wheat, rye, barley, oats, or their crossbred varieties, which have been specially processed to reduce the gluten content, contains no more than 100 mg/kg of gluten in the food as sold to the final consumer [10]. According to the painful limit of exposure, the serving portion of these products, and the quantity of gluten found, Table 4 shows examples of the potential allergen intake through the consumption of these products. It must be remarked that, in most of them, the estimated intake doses exceeded the limit of 10 mg in a serving portion.

The use of certain types of allergen-related labeling claims to attract consumers is a widespread practice among manufacturers, but a lack of control over potential allergens is often observed. In the case of patients allergic to milk or eggs, “vegan” products, such as soy or oat beverages, represent a suitable and safe alternative, such that they are frequently consumed by them. However, the results exposed that 64.9% of the notifications related to these types of products included undeclared allergens of animal origin (milk or eggs). The results are in accordance with those founded by Bedford et al. (2017). In their study, the authors observed that 50% of chocolates labeled “dairy-free” or “lactose-free”, as well as 25% of those labeled “vegan”, tested positive for milk, all with concentrations >1000 ppm [27].

Regarding milk as an allergen, current legislation only considers it mandatory to highlight the term “milk” to clearly differentiate it from the rest of a list of ingredients; however, it would be appropriate to review this legislation. When possible, labels should discern between the presence of lactose and milk proteins, instead of using only the term “milk” without more precise details; doing so would provide more practical information to patients, allowing them to make more convenient purchase choices according to their pathology, whether that be a milk protein allergy, lactose intolerance, or even if they are galactosemic. This discernment should also be systematically implemented in RASFF notifications to provide more accurate information for those patients. Although it varies a great deal according to the individual, as is the case with all food allergens, the threshold dose that induces symptoms in 5% of patients allergic to milk is less than 30 mg of milk proteins [28]. This low threshold dose implies an important risk for allergic consumers. It should also be remarked that, according to this symptomatic dose and the range of undeclared milk detected, a serving portion of 200 g of the product could imply, in some cases, a lactoprotein intake of 500 mg, almost 17 times higher than the dosage limit. 

In contrast, regarding lactose, the EFSA Panel on Dietetic Products, Nutrition and Allergies concluded that symptoms of lactose intolerance have been described after the intake of less than 6 g of lactose in some subjects. However, the vast majority of subjects with lactose maldigestion could tolerate up to 12 g of lactose as a single dose with no (or only minor) symptoms. Additionally, it has been concluded that higher doses might be tolerated if they are distributed throughout the day [29]. It is relevant that even the highest amounts of undeclared lactose found (1.56 g/100 g) do not exceed the tolerance limit for intolerant patients. However, undeclared lactose also implies a serious risk for patients with galactosemia. For them, the threshold dose of lactose and galactose is lower, and strict lactose avoidance is required. It has been suggested to disallow all foods with a galactose content of >20 mg/100 g [30]. With all of this being the case, many of the undeclared lactose products about which notifications were emitted represented a severe danger for these patients.

In EU legislation, the claim “lactose-free” has only been defined for infant and follow-on formula (≤10 mg/100 Kcal). Notwithstanding, some EU member states have set threshold levels at the national level for the use of the terms “lactose-free” or “low lactose” for foodstuffs other than those intended for infants. Unfortunately, a common level has not been adopted among these EU member states, and the lactose threshold level in “lactose-free” products varies between “absence of lactose and galactose” and 100 mg/100 g of the final product [29]. The lack of a common criterion adopted by all EU member states causes management and trade between countries difficult for manufacturers and can easily lead to notifications being emitted.

On the other hand, vegan patterns have globally risen during the last several years, including in FA pediatric patients [31,32]. Among the different motivations of plant-based dieters, the aversion to animal products due to moral and ethical reasons is highlighted [33]. In this context, the presence of undeclared allergens of animal origin, such as eggs, milk, fish, or seafood, in “vegan-claimed products” threatens the freedom of choice of this population and may pose a moral challenge to them.

Cross-reactivity is another problem of undeclared allergens in products. For example, a 75% cross-reactivity between soy as a primary food allergy and peanuts as a cross-reactive food has been observed [34]. If an allergic consumer were to ingest a product free of the primary trigger allergen, but it contains the cross-reactive undeclared allergen, it could imply a risk for their health.

The fact that 51.1% of the notifications of undeclared allergens had their origin in companies themselves highlights the efforts made by operators to control allergens. However, the proper management of allergens in the food industry implies a great challenge and a cost increase for them. Gupta et al. (2017) estimated this production cost increase as being between 10 and 30% [35]. To control this hazard, manufacturers routinely develop and implement independent allergen control plans to minimize the risk of product contact with food allergen contaminants and prevent recall events due to undeclared allergens. These plans typically specify practices for the safe handling and storage of raw materials, employee training, facility and equipment design, cleaning procedures, and production scheduling. Recalls due to food allergen cross-contact, cleaning procedures, equipment and premises design, and employee training were ranked by companies as the greatest allergen management expenses. In addition, companies may use precautionary allergen labeling (PAL), such as “may contain” on packaging, to label products for which there is a risk of cross-contact with food allergens during production. However, PAL usage remains voluntary and unregulated, and it currently presents consumers with considerable challenges due to its inconsistent use. The European Commission should also direct its efforts to address the inconsistent usage of PALs, promoting the harmonization of language used in PALs, and improving PAL status to quantified PAL statements, as previously suggested by other authors [36]. It would be helpful in communicating risks for both manufacturers and consumers, so that they can make informed choices when purchasing food products.

Food producers must continue increasing their efforts to improve food allergen management in order to reduce the presence of undeclared allergens in their products. For example, to reach this objective, they can follow and apply available guidance, such as *Guidance on Food Allergen Management for Food Manufacturers* [37]. Furthermore, it would also be highly recommended to implement international standards, such as voluntary food security and quality certifications, for example, International Food Standards version 6.1 [38] or BRGCS’s Gluten-Free Certification Program (GFCP) [39]. Currently, scientific advances have allowed the development of technological alternatives, such as irradiation or high hydrostatic pressure, in which, in addition to preserving nutrients, freshness, and organoleptic characteristics, they alter the structure of the proteins causing allergies, reducing their allergenicity [40,41]. However, these kinds of technologies are still under development, and their costs are very high, so they cannot be used by many companies. In the current scene, food manufacturers should improve food allergen management in their practices by focusing on empowering employees through more knowledge about food allergens and allergies, as well as through the use of new digital tools such as big data, as previously proposed by Jia and Evans (2021) [42].

The management of food allergies and dietary avoidance presents several challenges for pediatric dietitians and other healthcare providers [43]. Health professionals who assess FA patients should include nutrition therapy to ensure the adequate intake of nutrients as well as nutritional education with comprehensive information about allergenic ingredients for their avoidance [31,44]. The American Academy of Pediatrics established as a critical issue the improving of the education and training of all stakeholders for recognizing and managing, as well as preventing, allergic reactions. This work includes updating, creating, and implementing various guidelines and educational programs [45]. In this line, educational programs should be based on three basic aspects:

1. Learning which foods, due to their composition, may contain the trigger allergen. 

2. Understanding how to read labels to find out if the trigger allergen is present, including “contains traces of” or “may contain” statements. In this sense, it is also essential to note that unpackaged products, packaged directly in store or those sold in the form of self-service (e.g., bread and pastries offered in supermarkets) are very prone to cross-contamination with various allergens. However, despite it being mandatory to inform customers about the allergens present, the absence of a label on products and the high risk of cross-contamination cause their consumption by allergic patients to be unrecommended. 

3. Learning resources containing information about undeclared allergens in food labeling, as well as those products more likely to contain them according to the trigger allergen. On this point, it would be useful to provide information about official resources and webpages that share food allergen alerts, such as RASFF.

It would be helpful to provide patients with summarized and visual information about those products that most frequently present undeclared allergens, such as that shown in Table 5.

As it has been demonstrated that labeling is not a sufficient guarantee, and that there may be undeclared allergens, a useful reference tool for allergic patients and their families is the RASFF consumers’ portal. Launched in June 2014, this website is a consumer-friendly internet tool that provides the latest information on food recall notices. It also includes public health warnings issued by food safety authorities and food companies. By using this free tool, EU consumers could identify food that has been flagged in the system, allowing them to make more safety choices. There, users obtain access to practical information on product recalls and public health warnings in any given EU country. Additionally, they could also consult the institutional websites of the member states. The RASFF consumers’ portal and most websites provide food alert information on any food hazards, including undeclared allergens. Some of them are shown in Table 6.

In this respect, free mobile applications, such as HRana from the Croatian Ministry of Agriculture, are very useful for consumers in general, and for allergic patients in particular. This application enables citizens to receive information within 24 h on warnings regarding food, animal feed, and objects, as well as materials that come into direct contact with food which are sold in the Croatian and/or EU markets. Information that the Ministry of Agriculture updates in real time is sent to citizens via an application notification that displays food-related warnings and additional information on the non-compliant product (product name and image, shelf life, type of health risk, action taken by competent institutions, distribution status, etc.), as well as information on the food business operator that markets the product [60].

## 5. Conclusions

The results of this study reinforce the evidence of the risk associated with the presence of undeclared allergens on food labels for allergic patients. Of particular concern is the undeclared presence of those allergens with the highest prevalence in the pediatric population, in foods frequently consumed by this population, such as cereal and bakery products, prepared dishes and snacks, and cocoa and confectionery. Manufacturers and safety authorities should increase their efforts to face this risk, and educational programs could enforce patients’ knowledge about potential, undeclared-allergen food products with official resources and webpages, such as the RASFF consumers’ portal, which share updated food allergen alerts.

## Figures and Tables

**Table 1:** Mandatory declaration substances or products causing allergies or intolerances in the European Union according to EU Regulation 1169/2011 [7].

Substances or Products Causing Allergies or Intolerances	Exceptions
- Cereals containing gluten, namely wheat, rye, barley, oats, spelt, kamut, or their hybridized strains, and products thereof	- Wheat-based glucose syrups, including dextrose - Wheat-based maltodextrins - Glucose syrups based on barley - Cereals used for making alcoholic distillates, including ethyl alcohol, of agricultural origin
- Crustaceans and products thereof	
- Eggs and products thereof	
- Fish and products thereof	(a) Fish gelatin used as a carrier for vitamin or carotenoid preparations (b) Fish gelatin or isinglass used as a fining agent in beer and wine
- Peanuts and products thereof	
- Soybeans and products thereof	(a) Fully refined soybean oil and fat (b) Natural, mixed tocopherols (E306); natural D-alpha tocopherol; natural D-alpha tocopherol acetate; and natural D-alpha tocopherol succinate from soybean sources (c) Vegetable-oil-derived phytosterols and phytosterol esters from soybean sources (d) Plant stanol ester produced from vegetable-oil sterols from soybean sources
- Milk and products thereof (including lactose)	- Whey used for making alcoholic distillates, including ethyl alcohol, of agricultural origin - Lactitol
- Nuts, namely almonds (*Amygdalus communis* L.), hazelnuts (*Corylus avellana*), walnuts (*Juglans regia*), cashews (*Anacardium occidentale*), pecans (*Carya illinoinensis* (Wangenh.) K. Koch), Brazil nuts (*Bertholletia excelsa*), pistachio nuts (*Pistacia vera*), macadamia or Queensland nuts (*Macadamia ternifolia*), and products thereof, except for nuts used in making alcoholic distillates, including ethyl alcohol, of agricultural origin	
- Celery and products thereof	
- Mustard and products thereof	
- Sesame seeds and products thereof	
- Sulfur dioxide and sulfites at concentrations of more than 10 mg/kg or 10 mg/L in terms of the total SO_2_, which are to be calculated for products proposed as ready for consumption or as reconstituted according to the instructions of the manufacturers	
- Lupin and products thereof	
- Mollusks and products thereof	

**Table 2:** Percentage of RASFF notifications that informed about food products that simultaneously contained two or more undeclared allergens between 1 January 2018 and 31 December 2021.

Number of Allergens Present	% of Notifications
Two undeclared allergens	62.0
Three undeclared allergens	24.1
Four undeclared allergens	4.4
Five undeclared allergens	8.0
Six undeclared allergens	1.5

**Table 3:** Percentage of notifications which included specially designed products (gluten-free, lactose-free, dairy-free, etc.) and undeclared allergens involved in each case between 1 January 2018 and 31 December 2021.

Label Declaration	Total Notifications (%)	Undeclared Allergens	Examples of Notification-Related Products
“Gluten-free”	5.9	Gluten, lactose, milk, soy, and nuts	Organic, gluten-free, chocolate-coated, crispy cereals; gluten-free bread; and gluten-free pasta
“Dairy-free”	0.6	Milk, nuts, and soy	Chilled, dairy-free, coconut milk yogurt
“Lactose-free”	0.4	Milk, lactoprotein, lactose, and gluten	Lactose-free biscuits with buckwheat and chocolate
“Lactose-free and gluten-free”	0.4	Milk, lactoprotein, nuts, and soy	Gluten- and lactose-free chocolate spread
“Milk- and gluten-free”	0.2	Gluten, milk	Milk- and gluten-free vegan choco dessert with coconut cream
“Lactose-free” and “milk-protein-free”	0.1	Milk	Lactose- and milk-protein-free cake cream

**Table 4:** Examples of estimated gluten-intake dose via “gluten-free” products according to their serving portions and notifications of undeclared gluten amounts.

Product Claimed to be “Gluten-Free”	Quantity of Undeclared Gluten (mg/Kg—ppm)	Serving Portion (g)	Estimated Gluten Intake Dose (mg/Serving Portion)
Gluten-free corn pasta	176	150	26.4
Gluten-free cream preparation with potatoes and leeks	31.6	210	6.6
Gluten-free pasta	75.3	150	1.3
Gluten-free corn chips	265	30	8.0
Gluten-free peanut butter protein bar	853	40	34.1
Gluten-free hummus	>150	100	15.0
Gluten-free hemp protein	930	30	27.9
Gluten-free buckwheat and sweet potato noodles	>80	150	1.0
Gluten-free yellow lentil lasagna and spaghetti	88	200	17.6
Gluten-free vegetarian nuggets	38	100	3.8

**Table 5:** Summary of the main allergens that affect the pediatric population, and main risk food category.

Allergen	Notified Food Category	Notified Food Examples
Milk (lactoproteins and lactose)	Cereal and bakery products	Organic rice pancakes with dark chocolate, biscuits, and popcorn
	Prepared dishes and snacks	Sausage rolls, grilled chips, and packed snacks
Nuts	Cereal and bakery products	Pesto-and-walnut-plucked bread, chocolate pie, and energy cereal bars
	Nuts, nut products, and seeds	Pistachio cream; fruit–nut mix; and peanut, nut, and mulberry mix
	Cocoa and cocoa preparations	Milk chocolate with buckwheat; chocolate; and chocolate spread
Gluten	Cereal and bakery products	Rice-flour cake mix, sugar loaves, bulgur and pasta, and hot dog buns
	Prepared dishes and snacks	Rice salad, baby food, and tortilla chips
Egg	Cereal and bakery products	Donuts with cocoa coating, red velvet muffins, and bakery scones
	Prepared dishes and snacks	Hummus and aioli, pizza, and prepared sandwiches
Soya	Cereal and bakery products	Rice flour, cheese and onion bread, and butter croissants
	Prepared dishes and snacks	Beef-flavored instant rice noodles, nacho-cheese snacks, and potato chips
Peanut	Nuts, nut products, and seeds	Almond paste; nut mix; and roasted, organic, almond kernels
	Confectionery	Baklava, wafer rolls with cream, and candies
Fish	Dietetic foods, food supplements, and fortified foods	Food supplements and organic infant milk starter packs
	Prepared dishes and snacks	Club-salad pasta, tuna and chicken, chilled tandoori chicken salad dishes, and salmon lasagna
Seafood (mollusks and crustaceans)	Dietetic foods, food supplements, and fortified foods	Food supplements
	Prepared dishes and snacks	Frozen cheese croquettes; frozen lamb and carrot dumplings; and chilled, spicy chicken salad

**Table 6:** Examples of institutional websites of European Union countries that provide practical undeclared allergen information (product recalls and other public health warnings).

Country	Institution/ Public Organism	Website	
European Commission	RASFF consumers’ portal	https://webgate.ec.europa.eu/rasff-window/screen/consumers (accessed on 10 March 2022)	[14]
Austria	Austrian Agency for Health and Food Safety (AGES)	https://www.ages.at/en/human/product-warnings-product-recalls (accessed on 10 March 2022)	[46]
Belgium	Federal Agency for the Safety of the Food Chain (AFSCA)	https://www.favv-afsca.be/consommateurs/avertissements/ (accessed on 10 March 2022)	[47]
Croatia	Government of the Republic of Croatia	https://gov.hr/en/hrana-food-related-warnings/1774 (accessed on 10 March 2022)	[48]
France	Ministry of Agriculture and Food	https://rappel.conso.gouv.fr/categorie/1#navigation (accessed on 10 March 2022)	[49]
Germany	Federal Office of Consumer Protection and Food Safety	https://www.lebensmittelwarnung.de/bvl-lmwde/liste/lebensmittel/deutschlandweit/10/0 (accessed on 10 March 2022)	[50]
Greece	Hellenic Food Authority (EFET)	https://www.efet.gr/index.php/en/ (accessed on 10 March 2022)	[51]
Ireland	Food Safety Authority of Ireland	https://www.fsai.ie/ (accessed on 10 March 2022)	[52]
Italy	Ministry of Health	https://www.salute.gov.it/portale/news/p3_2_1_3.jsp?lingua=italiano&menu=notizie&p=avvisi (accessed on 10 March 2022)	[53]
Luxembourg	Food safety—Grand Duchy of Luxembourg	https://securite-alimentaire.public.lu/fr.html (accessed on 10 March 2022)	[54]
Malta	Environmental Health Directorate	https://deputyprimeminister.gov.mt/en/environmental/Pages/Home-Page.aspx (accessed on 10 March 2022)	[55]
Netherlands	Netherlands Food and Consumers Product Safety Authority	https://www.nvwa.nl/onderwerpen/veiligheidswaarschuwingen (accessed on 10 March 2022)	[56]
Romania	National Sanitary Veterinary and Food Safety Authority (ANSVSA)	http://www.ansvsa.ro/informatii-pentru-public/produse-rechemateretrase/ (accessed on 10 March 2022)	[57]
Slovenia	Republic of Slovenia	https://www.gov.si/drzavni-organi/organi-v-sestavi/uprava-za-varno-hrano-veterinarstvo-in-varstvo-rastlin/ (accessed on 10 March 2022)	[58]
Spain	Spanish Agency for Food Safety and Nutrition (AECOSAN)	https://www.aesan.gob.es/AECOSAN/web/subhomes/seguridad_alimentaria/aecosan_seguridad_alimentaria.htm (accessed on 10 March 2022)	[59]

## Supplementary Materials

The following are available online at https://www.mdpi.com/article/10.3390/nu14081571/s1. Table S1: Food products included per food category in the RASFF notifications from 2018 to 2021.

## Appendix A

**Table A1:** Food products involved in “other/mixed products category” in the RASFF notifications from 1 January 2018 and 31 December 2021.

Year 2018	Year 2019	Year 2020	Year 2021
Aubergines in oil	Carbonated strawberry-and-cream-flavored soft drink	Sweet and sour chicken	Canned soybeans mislabeled as spelt
Cake cream labelled as “lactose-free” and “milk-protein-free”	Carrot and dill hummus		Chilled, smoked-salmon salads
Canned, peeled tomatoes	Chilled Hawaiian chicken salad	Frozen, organic, mung-bean nuggets	Chilled strawberries with chocolate cream
Chips	Frozen garlic puree	Frozen spring-roll sheets	Chilled vegan paprika slices
Cooked white beans	Frozen potato waffles	Frozen veggie burgers	Croutons used in Caesar salad
Easter-egg dye	Frozen soya product	Melba toast	Frozen beef spring rolls
Gluten-free bread mix	Frozen spicy burgers	Organic coconut sugar	Frozen sandwiches
Gluten-free-protein linseed and protein powder	Gluten-free hemp protein	Roasted vegetables	Grilled bell-pepper tapenade
Grilled chicken dish and tuna salad	Gluten-free hummus	Spinach noodles	Olives
Mini mooncakes	Green olives in glass jars	Various frozen products	Organic, gluten-free, soy flour
Organic, veggie-style chicken chunks mislabeled and packed as veggie-style beef strips	Grilled artichokes in sunflower oil	Vegan pate	Organic hummus
Pickled pepperoni	Instant pumpkin cereal	Vegetarian, marinated fillet	Pink roses for cake decoration
Various foodstuffs	Mini vegetarian-burger nuggets		Spaghetti Bolognese
Various halawa flavors and jams	Olive paste		Spicy peppers filled with tuna and capers
Vegan, dairy-free, grated pizza topping	Organic powder preparation for fermented soy dessert		Spinach cream
Vegan, stracciatella-flavored, lupin-based yogurt	Pasta salad with smoked salmon		Spreads
Wafer sheets	Seitan and vegetable sausages		Various gluten-free, processed products
	Soy-meat products		Vegetarian burgers
	Spicy carrot spread with ginger		Vegetarian sausages
	Sugar-free licorice sweets		
	Vegetable snack		
	Vegetarian mince mistakenly packaged as roasted cubes		
	Vegetarian salami		

## References

[1] Canonica G.W., Holgate S., Lockey R., Pawankar R., World Health Organization (2013). White Book on Allergy 2011–2012 Executive Summary.

[2] Loh W., Tang M.L.K. (2018). The Epidemiology of Food Allergy in the Global Context. Int. J. Environ. Res. Public Health.

[3] Lyons S.A., Clausen M., Knulst A.C., Ballmer-Weber B.K., Fernandez-Rivas M., Barreales L., Bieli C., Dubakiene R., Fernandez-Perez C., Jedrzejczak-Czechowicz M. (2020). Prevalence of Food Sensitization and Food Allergy in Children across Europe. J. Allergy Clin. Immunol. Pract..

[4] Sicherer S.H., Sampson H.A. (2013). Food Allergy: Epidemiology, Pathogenesis, Diagnosis, and Treatment. J. Allergy Clin. Immunol..

[5] Nwaru B.I., Hickstein L., Panesar S.S., Roberts G., Muraro A., Sheikh A. (2014). Prevalence of Common Food Allergies in Europe: A Systematic Review and Meta-Analysis. Allergy.

[6] Agache I. (2018). EAACI White Paper on Research, Innovation and Quality Care.

[7] The European Parliament and the Council of the European Union (2011). Regulation (EU) No 1169/2011 of the European Parliament and of the Council of 25 October 2011 on the Provision of Food Information to Consumers, Amending Regulations (EC) No 1924/2006 and (EC) No 1925/2006 of the European Parliament and of the Council, and Repealing Commission Directive 87/250/EEC, Council Directive 90/496/EEC, Commission Directive 1999/10/EC, Directive 2000/13/EC of the European Parliament and of the Council, Commission Directives 2002/67/EC and 2008/5/EC and Commission Regulation (EC) No 608/2004 Text with EEA Relevance. Off. J. Eur. Union.

[8] EFSA NDA Panel (EFSA Panel on Dietetic Products, Nutrition and Allergies) (2014). Scientific Opinion on the Evaluation of Allergenic Foods and Food Ingredients for Labelling Purposes. EFSA J..

[9] (2015). Royal Decree 126/2015 of 27 February 2015, which Approves the General Rule on Food Information on Foodstuffs Presented Unpackaged for Sale to the Final Consumer and to Mass Caterers, those Packaged at the Point of Sale at the Request of the Purchaser, and those Packaged by Retail Trade Operators. BOE.

[10] (2014). Commission Implementing Regulation (EU) no 828/2014 of 30 July 2014 on the Requirements for the Provision of Information to Consumers on the Absence Or Reduced Presence of Gluten in Food (Text with EEA Relevance). Off. J. Eur. Union.

[11] (2016). Commission Delegated Regulation (EU) 2016/127 of 25 September 2015 Supplementing Regulation (EU) no 609/2013 of the European Parliament and of the Council as Regards the Specific Compositional and Information Requirements for Infant Formula and Follow-on Formula and as Regards Requirements on Information Relating to Infant and Young Child Feeding (Text with EEA Relevance). Off. J. Eur. Union.

[12] AESAN Notice of 26 July 2019 Relating to the Conditions of use of the Terms ‘Lactose-Free’ and ‘Low Lactose’. https://www.aesan.gob.es/AECOSAN/docs/documentos/seguridad_alimentaria/interpretaciones/nutricionales/sin_lactosa.pdf.

[13] Soon J.M., Abdul Wahab I.R. (2021). Global Food Recalls and Alerts Associated with Labelling Errors and its Contributory Factors. Trends Food Sci. Technol..

[14] RASFF Window Version 2.1.0. European Commission. https://webgate.ec.europa.eu/rasff-window/screen/search.

[15] The Official Portal for European Data European Commission. https://data.europa.eu/en.

[16] Peters R.L., Krawiec M., Koplin J.J., Santos A.F. (2021). Update on Food Allergy. Pediatr. Allergy Immunol..

[17] Fleischer D.M., Perry T.T., Atkins D., Wood R.A., Burks A.W., Jones S.M., Henning A.K., Stablein D., Sampson H.A., Sicherer S.H. (2012). Allergic Reactions to Foods in Preschool-Aged Children in a Prospective Observational Food Allergy Study. Pediatrics.

[18] Turner P.J., Jerschow E., Umasunthar T., Lin R., Campbell D.E., Boyle R.J. (2017). Fatal Anaphylaxis: Mortality Rate and Risk Factors. J. Allergy Clin. Immunol. Pract..

[19] Baseggio Conrado A., Patel N., Turner P.J. (2021). Global Patterns in Anaphylaxis due to Specific Foods: A systematic Review. J. Allergy Clin. Immunol..

[20] Turner P.J., Gowland M.H., Sharma V., Ierodiakonou D., Harper N., Garcez T., Pumphrey R., Boyle R.J. (2014). Increase in Anaphylaxis-Related Hospitalizations but no Increase in Fatalities: An Analysis of United Kingdom National Anaphylaxis Data, 1992–2012. J. Allergy Clin. Immunol..

[21] Kleter G.A., Prandini A., Filippi L., Marvin H.J.P. (2009). Identification of Potentially Emerging Food Safety Issues by Analysis of Reports Published by the European Community’s Rapid Alert System for Food and Feed (RASFF) during a Four-Year Period. Food Chem. Toxicol..

[22] Soon J.M., Brazier A.K.M., Wallace C.A. (2020). Determining Common Contributory Factors in Food Safety Incidents—A Review of Global Outbreaks and Recalls 2008–2018. Trends Food Sci. Technol..

[23] Gendel S.M., Jianmei Z. (2013). Analysis of U.S. Food and Drug Administration Food Allergen Recalls After Implementation of the Food Allergen Labeling and Consumer Protection Act. J. Food Prot..

[24] Bucchini L., Guzzon A., Poms R., Senyuva H. (2016). Analysis and Critical Comparison of Food Allergen Recalls from the European Union, USA, Canada, Hong Kong, Australia and New Zealand. Food Addit. Contam. Part A Chem. Anal. Control Expo. Risk Assess..

[25] Hischenhuber C., Crevel R., Jarry B., Maki M., Moneret-Vautrin D.A., Romano A., Troncone R., Ward R. (2006). Review Article: Safe Amounts of Gluten for Patients with Wheat Allergy Or Coeliac Disease. Aliment Pharmacol. Ther..

[26] Akobeng A.K., Thomas A.G. (2008). Systematic Review: Tolerable Amount of Gluten for People with Coeliac Disease. Aliment Pharmacol. Ther..

[27] Bedford B., Yu Y., Wang X., Garber E.A.E., Jackson L.S. (2017). A Limited Survey of Dark Chocolate Bars obtained in the United States for Undeclared Milk and Peanut Allergens. J. Food Prot..

[28] Moneret-Vautrin D.A., Kanny G. (2004). Update on Threshold Doses of Food Allergens: Implications for Patients and the Food Industry. Curr. Opin. Allergy Clin. Immunol..

[29] EFSA Panel on Dietetic Products, Nutrition and Allergies (NDA) (2010). Scientific Opinion on Lactose Thresholds in Lactose Intolerance and Galactosaemia. EFSA J..

[30] Gropper S., Weese J., West P., Gross K. (2000). Free Galactose Content of Fresh Fruits and Strained Fruit and Vegetable Baby Foods:More Foods to Consider for the Galactose-Restricted Diet. J. Acad. Nutr. Diet..

[31] Protudjer J.L.P., Mikkelsen A. (2020). Veganism and Paediatric Food Allergy: Two Increasingly Prevalent Dietary Issues that are Challenging when Co-Occurring. BMC Pediatr..

[32] Sutter D.O., Bender N. (2021). Nutrient Status and Growth in Vegan Children. Nutr. Res..

[33] Rosenfeld D.L., Burrow A.L. (2017). Vegetarian on Purpose: Understanding the Motivations of Plant-Based Dieters. Appetite.

[34] Cox A.L., Eigenmann P.A., Sicherer S.H. (2021). Clinical Relevance of Cross-Reactivity in Food Allergy. J. Allergy Clin. Immunol. Pract..

[35] Gupta R.S., Taylor S.L., Baumert J.L., Kao L.M., Schuster E., Smith B.M. (2017). Economic Factors Impacting Food Allergen Management: Perspectives from the Food Industry. J. Food Prot..

[36] Soon J.M., Manning L. (2017). “May Contain” Allergen Statements: Facilitating Or Frustrating Consumers?. J. Consum. Policy.

[37] Food Drink Europe (2013). Guidance on Food Allergen Management for Food Manufacturers.

[38] IFS Management GmbH (2017). IFS Food Version 6.1..

[39] BRCGS (2019). Gluten-Free Certification Program Global Standard (Issue 3).

[40] Zhang Y., Ren Y., Bi Y., Wang Q., Cheng K., Chen F. (2019). Review: Seafood Allergy and Potential Application of High Hydrostatic Pressure to Reduce Seafood Allergenicity. Int. J. Food Eng..

[41] Pan M., Yang J., Liu K., Xie X., Hong L., Wang S., Wang S. (2021). Irradiation Technology: An Effective and Promising Strategy for Eliminating Food Allergens. Food Res. Int..

[42] Jia L., Evans S. (2021). Improving Food Allergen Management in Food Manufacturing: An Incentive-Based Approach. Food Control.

[43] Groetch M.E., Christie L., Vargas P.A., Jones S.M., Sicherer S.H. (2010). Food Allergy Educational Needs of Pediatric Dietitians: A Survey by the Consortium of Food Allergy Research. J. Nutr. Educ. Behav..

[44] Collins S.C. (2016). Practice Paper of the Academy of Nutrition and Dietetics: Role of the Registered Dietitian Nutritionist in the Diagnosis and Management of Food Allergies. J. Acad. Nutr. Diet..

[45] Sicherer S.H., Allen K., Lack G., Taylor S.L., Donovan S.M., Oria M. (2017). Critical Issues in Food Allergy: A National Academies Consensus Report. Pediatrics.

[46] Austrian Agency for Health and Food Safety (AGES) Österreichische Agentur für Gesundheit und Ernährungssicherheit GmbH. https://www.ages.at/en/human/product-warnings-product-recalls.

[47] AFSCA Pour Les Consommateurs Agence Fédérale Pour La Sécurité De La Chaîne Alimentaire. Federal Agency for the Safety of the Food Chain. https://www.favv-afsca.be/consommateurs/avertissements/.

[48] E-Citizens Information and Service HRana—Food-Related Warnings. Government of the Republic of Croatia. https://gov.hr/en/hrana-food-related-warnings/1774.

[49] Le Site Des Alertes De Produits Dangereux Ministry of Agriculture and Food. France Government. https://rappel.conso.gouv.fr/categorie/.

[50] Lebensmittelwarnung Das Bundesamt für Verbraucherschutz und Lebensmittelsicherheit, BVLD; Deutschland. www.lebensmittelwarnung.de.

[51] Recent News Hellenic Food Authority (EFET). Hellenic Food Authority. https://www.efet.gr/index.php/en/.

[52] Latest Food Alerts Food Safety Authority of Ireland. https://www.fsai.ie/.

[53] Avvisi Di Sicurezza. Sicurezza Alimentare. Ministero della Salute. Governo Italiano. https://www.salute.gov.it/portale/news/p3_2_1_3.jsp?lingua=italiano&menu=notizie&p=avvisi.

[54] Alertes Alimentaires Sécurité Aimentaire. Le Gouvernement du Gran-Duché de Luxembourg. https://securite-alimentaire.public.lu/fr.html.

[55] Food Alerts Latest News. Environmental Health Directorate, Government of Malta. https://deputyprimeminister.gov.mt/en/environmental/Pages/Home-Page.aspx.

[56] Veiligheidswaarschuwingen Nederlandse Voedsel- en Warenautoriteit, (NVWA). https://www.nvwa.nl/onderwerpen/veiligheidswaarschuwingen/overzicht-veiligheidswaarschuwingen.

[57] Rechemare/Retragere Produse Alimentare Autoritatea Națională Sanitară Veterinară și pentru Siguranța Alimentelor, (ANSVSA); Guvernul României. http://www.ansvsa.ro/informatii-pentru-public/produse-rechemateretrase/.

[58] Obvestila Za Potrošnike Uprava za varno hrano, veterinarstvo in varstvo rastlin.; Republika Slovenija. https://www.gov.si/drzavni-organi/organi-v-sestavi/uprava-za-varno-hrano-veterinarstvo-in-varstvo-rastlin/.

[59] Alertas Alimentarias De Alérgenos Seguridad Alimentaria.Agencia Española de Seguridad Alimentaria y Nutrición. Ministerio de Consumo. Gobierno de España. https://www.aesan.gob.es/AECOSAN/web/subhomes/seguridad_alimentaria/aecosan_seguridad_alimentaria.htm.

[60] HRana—Food-Related Warnings Ministry of Agriculture. Government of the Republic of Croatia. https://hrana.mps.hr/HRana/.

