# Supplementary material for: The Risk of Undeclared Allergens on Food Labels for Pediatric Patients in the European Union
Source: Nutrients. 2022 Apr 10;14(8):1571. doi: 10.3390/nu14081571 (PMC9026746; doi:10.3390/nu14081571)
Supplement: Supplementary file 1 [file nutrients-14-01571-s001.zip › nutrients-1662207-supplementary.pdf]

Table S1. Food products included per food category in the RASFF notifications between 1st January 2018 and 31st December 2021.

Product category	2018	2019	2020	2021
**Alcoholic beverages**		Beer		Beer
		Liqueur		Gluten-free beer
**Bivalve molluscs and products**				Scallop paté labelled as fish
**Cephalopods and products thereof**			Frozen cleaned cuttlefish (Sepia officinalis)	
**Cereals and bakery products**	Almond-stuffed croissants sold as all butter croissants	Baked biscuits bar with hazelnuts	Bagel	Almond biscuits
	Bakery scones	Biscuits	Biscuits filled with cocoa cream	Amaranth wafers
	Buckwheat flour	Biscuits with hazelnut flavoured filling	Breakfast cereals	Baby cereal porridge
	Buckwheat taralli	Bread	Brioche buns	Biscuits
	Cacao and almond granola mislabelled as blueberry, date and cardamom granola	Bread improver - enzyme mix	Bulgur and pasta	Biscuits filled with vanilla-flavoured cream
	Cannoli	Brown linseed	Caramel flavoured chocolate pastries	Bread buns
	Chilled pastry (tompouce) mislabelled as apple and apricot cake	Buns incorrectly labelled as dark buns	Cereal balls covered with chocolate	Butter croissant
	Chocolate and praline biscuits	Chocolate silver pearls	Cheese and onion bread	Cake
	Chocolate cake	Corn muffins	Chilled appel pie	Chocolate chip cookies mix
	Chocolate cereal bars	Energy cereal bars	Chilled pie crust	Cookies
	Chocolate pie	Fine bakery	Chocolate biscuits	Corn crackers with legumes
	Frozen pizza	Frozen chocolate buns mistakenly packaged as croissants	Chocolate-coated cashew nuts labelled as chocolate-coated speculoos balls	Couscous
	Frozen strawberry cheese cake	Frozen gluten and milk free chocolate cake	Cookies	Crunchy cereal flakes and chocolate
	Frozen vegan banana bread	Fruit muesli	Donuts with cocoa coating	Gluten-free pink cookies
	Gluten-free cereal bars	Garlic bread slices	Gluten-free bread kit	Gluten-free bread
	Gluten-free corn crisps	Gluten-free cassava flour	Gluten-free buckwheat and sweet potato noodles	Gluten-free buckwheat flour

Gluten-free corn pasta	Gluten-free muffins	Gluten-free coconut, chocolate and orange- and blueberry and chocolate-flavoured oatmeal cookies	Gluten-free chapati breads
Gluten-free marble cake mix dia	Gluten-free oat flour	Gluten-free granola	Gluten-free white buckwheat flour
Gluten-free pasta	Gluten-free organic brown rice chips with red lentils	Multi fruit muesli	Noodles
Gluten-free peanut butter protein bar	Gluten-free pasta	Organic brioches	Organic gluten-free chocolate-coated crispy cereals
Lactose-free & gluten-free biscuits	Gluten-free plum cake with yogurt	Organic gluten free chickpea flour spirelli	Organic multigrain porridge for infants
Lactose-free biscuits with buckwheat and chocolate	Gluten-free yellow lentil lasagne and spaghetti	Pastries (chebakia)	Pasta
Oat flakes	Gluten-free yellow lentil spaghetti	Rice flour	Pesto and walnut plucked bread
Organic rice pancakes with black chocolate	Granola	Shortcake biscuit	Plain and filled crepes
Pizza and barbecue flavoured biscuits	Hot dog buns	Sponge cake	Quinoa and kale corn puffs
Rice flour cake mix	Ice waffles	Tempura powder	Quinoa puffs
Sesame seeds crispbreads incorrectly labelled as dark rye crispbreads	Jaffa cakes	Wheat gluten	Red velvet muffins
Speck and pepper flavoured croutons	Mince pies	Whole grain rice cakes with dark chocolate coating	Spelt crackers
Speculoos bars	Mixed sesame cookies		Spelt seeds crackers
Thai crispy rolls	Organic chocolate brownie		Strawberry-flavoured biscuits
Thai sweet cereals	Organic kamut biscuits with figs		Wafer rolls with hazelnut cream filling
Wafers with caramel and cocoa cream filling	Organic rice cakes with 'rice milk' chocolate coating		White fondant roll
Wheat flour	Organic spelt flour		
	Organic wheat gluten		
	Pastries		
	Popcorn		
	Porridge		
	Protein bars		
	Red quinoa		

		Sugar loaf		
		Wafers		
**Cocoa and cocoa preparations, coffee and tea**	Chocolate chip tea	Candied orange peels covered with dark chocolate	Chocolate	Cocoa cream with hazelnuts
	Dark chocolate	Chocolate bars	Chocolate bars with rum filling	Dark chocolate with hazelnuts incorrectly packaged as dark chocolate with raisins and almonds
	Dark chocolate with almonds	Chocolate mousse with chocolate balls	Chocolate easter eggs	Dark chocolates
	Infusion	Chocolate nonpareils	Chocolate spreads	Milk chocolate with buckwheat
	Milk chocolate spread	Chocolate spread	Dark chocolate	Organic vegan gluten-free crispy chocolate hearts
	Milk chocolate with almonds	Dark chocolate	Dark chocolate with orange peel	Vegan chocolate
		Gluten and lactose free chocolate spread	Dark chocolate with peppermint filling	
		Gluten-free ready-to-bake dough for chocolate fondants and lemon cakes	Flower shaped chocolate	
		Ground chocolate-flavoured coffee	Fruit tea	
		Hazelnut chocolate spread	Milk chocolate	
		Organic whole milk chocolate	Milk chocolate and hazelnut pralines	
		Umbrella-shaped chocolate	Vegan and gluten-free cocoa, caramel and sea salt chocolate	
		Vegan hazelnut chocolate bar		
**Confectionery**	Amarena cherries in dark chocolate	Baklava	Apricot and figs jam	Apricot jam
	Apricot jam	Biscuits	Blueberry truffle pralines	Candies
	Blueberry chews	Candies	Cacao and orange bar	Chocolates
	Chocolate bars	Caramel and peanut bars incorrectly packaged as chocolate and caramel bars	Candied cherries with chocolate coating	Confectionery
	Chocolate covered caramel waffle	Chocolate candies	Chocolate caramel shortcake bites	Dried fruit confectionery with walnuts
	Coffee on a stick	Chocolate cups	Chocolate-covered coffee beans	Figs jam
	Dairy-free raw cocoa candy bars	Chocolate pralines	Gluten-free sugar sprinkles	Gluten-free candies
	Dates balls	Cookies	Gummies	Hard sweets

	Dragees<br>Fig jam<br>Handmade loukoumi<br>Hazelnut cream<br>Jelly candies<br>Powder for gluten-free desserts<br>Pralines<br>Raisins covered with dark chocolate<br>Sesame halva with cocoa<br>Sesame paste with cocoa<br>Turkish delight (lemon, rose flavours)<br>Wafer biscuits	Cookies with hazelnuts with pieces of chocolate<br>Dark chocolate chips<br>Dark chocolate chunks<br>Hazelnut chocolate wafers<br>Organic brown coconut sugar<br>Strawberry jam<br>Wafer rolls with cream	Marzipan covered with chocolate<br>Milk chocolate bonbons with nougat filling<br>Salted caramel brownie protein snacks<br>Strawberry, pistachio and chocolate flavoured macarons<br>Tiramisu ice cream<br>Wafers covered with chocolate	Raspberry eyes cookies<br>Starch and sugar decorations for confectionery<br>Vegan chocolat bars with strawberry flavor<br>Vegan chocolate covered biscuit bar
**Crustaceans and products thereof**	Chilled crabs (Cancer pagurus)<br>Chilled red shelled shrimps (Aristaeomorpha foliacea)<br>Frozen cooked crab<br>Live crabs	Frozen shrimp bites with spinach	Chilled Norway lobster (Nephrops norvegicus)<br>Norway lobsters (Nephrops norvegicus)	Chilled shrimp salad mislabelled as ham salad<br>Canned crab meat<br>Frozen prawn (Litopenaeus vannamei) skewers<br>Frozen cooked peeled shrimps (Solenocera crassicornis)<br>Frozen Norway lobster tails<br>Frozen peeled deepwater rose shrimps (Parapenaeus longirostris)<br>Frozen peeled shrimps (Parapenaeus longirostris)<br>Frozen shrimps (Litopenaeus vannamei)<br>Ground and whole crayfish<br>Thawed cooked shrimps (Litopenaeus vannamai)

**Dietetic foods, food supplements, fortified foods**	Chocolate flavoured soy powder shakes<br>Food for sports people<br>Food supplement<br>Food supplements<br>Organic chlorella<br>Organic chlorella and spirulina powder<br>Organic chlorella capsules<br>Organic chlorella powder<br>Organic chlorella powder and tablets<br>Organic chocolate-flavoured protein powder<br>Organic gluten-free baby food<br>Organic spirulina powder<br>Protein flan preparations<br>Strawberry and blueberry flavoured vegan powder shakes<br>Vegan protein	Food supplement<br>Food supplement with Chlorella pyrenoidosa<br>Glucosamine sulphate<br>Gluten-free organic milk thistle extract<br>Organic infant milk starter pack<br>Soy protein powder<br>Vegan proteins mix for sports people	Food supplement<br>Food supplement for children<br>Organic corn, rice and tapioca cream<br>Organic spirulina tablets<br>Peanuts and fudge bar<br>Spirulina tablets	Food supplement<br>Protein bar
**Eggs and egg products**	Rgg premix	Frozen cheese omelette mislabelled as omelette with fine herbs	Whole egg powder	
**Fats and oils**		Dairy-free organic vegan butter alternative<br>Margarine	Mustard oil	Chilled vegan gluten- and<br>Peanut oil
**Fish and fish products**	Chilled salmon preparation	Frozen breaded anchovy fillets<br>Frozen fish balls<br>Frozen imitation crab sticks<br>Gluten-free breaded fish<br>Smoked sardines	Crab sticks<br>Frozen breaded fish fillets<br>Shrimp salad	Chilled breaded fish<br>Chilled sushi salmon wraps
**Food additives and flavourings**	Snowflake decoration	Whey powder incorrectly labelled as xanthan gum	Wasabi powder	Organic mushroom reishi powder
	Broccoli sprouts	Dried apricots	Apple and plum puree	Candied ginger

**Fruits and vegetables**	Dried appel rings and coconut flakes	Dried sweet potato	Banana in syrup	Candied mango with plum powder
	Dried apricots	Dried waxberries	Dried mushroom shiitake	Canned chickpeas in water
	Dried mangoes	Frozen Asian wok mix	Dried mushrooms	Chilled organic spinach burgers
	Frozen vegetables mix	Golden sultanas	Dried tomato chips	Chilled vegetable mix incorrectly packaged
	Lychees	Grated coconut	Edamame beans	Dried apples
	Organic asparagus salad	Mix of dried fruits	Frozen spinach	Dried figs
	Organic chlorella powder	Organic chlorella	Green olives stuffed with pepper	Dried fruits
	Organic white beans	Pickled lotus rootlets	Green sultanas	Dried mushrooms
	Palmyrah fruit pulp	Pickled mango slices	Parsley	Dried shiitake mushrooms
	Pickled lotus rootlets	Raisins	Pickled chopped cucumbers	Frozen mixed vegetables
	Preserved lemons	Several vegetable products	Pickled eggplants	Frozen spinach leaves and creamed spinach
	Sauerkraut	Silver onions used in salads	Preserved artichoke hearts	Frozen vegetables
	Sweet potato fries		Raisins	Green pitted olives
	Vegetable medley		Yellow raisins	Green raisins
				Lychees
				Mixed pickles
				Organic strawberry fruit spread
				Pickled turnips
				Shiitake mushrooms
				Sterilized vegetables
				Sundried tomatoes
**Herbs and spices**	Curry powder	Aromatic spice blend	Ground cumin	Cinnamon
	Ground nutmeg	Curry mix	Thyme spice mix	Coriander seeds
	Ground white pepper	Curry powder		Dried lily bulbs
	Masala spices mix	Ground cinnamon		Ground cumin
	Spice mixture Ras El Hanout	Ground mustard		Herb salt
	White pepper	Mixed spices for hamburger		Madras curry
		Pizza spice		Mild curry powder

		Spice mix		Seasoning salt
		Spices		Spice mix
**Honey and royal jelly**	natural polyfloral honey			
**Ices and desserts**	Chilled paste for the production of ice cream	Chilled rice dessert with raspberry flavour	Caramel coconut flavoured ice cream	Chocolate roses
	Chocolate ice cream	Hazelnut stracciatella ice cream	Chilled vegan organic chocolate-flavoured hemp desserts	Chocolate-coated groundnuts ice cream
	Gluten-free ice cream	Ice cream and dessert drinks	Chocolate fondant	Organic ice cream
	Ice cream	Lemon cremeux meringue's crumble	Ice cream	
	Ice cream cones	Milk and gluten-free vegan choco dessert with coconut cream	Pastries	
	Soy vegan ice cream cones with vanilla flavour	Non-dairy ice cream	Premix for preparation of ice cream	
	Vanilla and chocolate ice cream dessert covered with chocolate and cereals	Soft marshmallow with chocolate coating		
		Strawberry ice cones		
		Vanilla ice cream with chocolate		
		Vegan chocolate and coconut milk ice cream		
		Vegan dark chocolate mousse		
		Vegetarian vanilla ice cream		
**Meat and meat products (other than poultry)**	Bacon	Chilled beef roast	Chilled gluten-free angus beef burgers	Chilled cranberry paté
	Chilled cheese sausages labelled as grill sausages	Chilled burgers	Chilled pork medallions	Chilled meatballs
	Frozen roasted cantonese-style pork (cha siu)	Chilled meat preparation	Chilled sliced meat loaf with leeks	Cured pork loins
	Ham with nuts	Chilled meat ragout	Frozen grilled gyros	Frozen pig meat preparation
		Chilled pork sausage	Frozen minced meat patties	Frozen sliced and roasted beef kebab
		Chilled sausages	Frozen Taiwanese braised minced pork	
		Chilled stew meat	Kebab	

		Cold pork luncheon meat<br>Frozen meatballs<br>Incorrectly labelled pate<br>Liver pate<br>Salami<br>Sausages<br>Walnut in sausages with walnuts	Pate<br>Pork pate<br>Sausages	
**Milk and milk products**	Lactose-free milk<br>Yoghurt with muesli and raspberry sauce	Acidified semi-skimmed milk<br>Cheese<br>Frozen camembert bites<br>Yogurt based sauce incorrectly labelled as humus	Almond flavoured ice cream<br>Cheese<br>Chilled cheeses<br>Fruit sherbets<br>Lactose-free whipped cream<br>Lactose-free yogurt<br>Organic halloumi<br>Pasteurized cow's milk cream cheese<br>Skimmed yoghurt drink with red berries<br>Spicy cheese	Butter<br>Chocolate sponge cake with milk and coconut filling<br>Grana padano cheese<br>Grated goat cheese<br>Mustard cheese<br>Yoghurt
**Non-alcoholic beverages**	Vegetable drink	Chilled mango and oatmeal smoothie	Carbonated strawberry and cream flavoured soft drink<br>Green juice drink<br>Mango drink	Vegetable juice
**Nuts, nut products and seeds**	Almond powder<br>Canned coconut cream<br>Cheese and bacon cocktail nuts<br>Chilled dairy-free coconut milk yogurt<br>Coconut milk for cooking	Chilli cashew & groundnut mix<br>Coconut milk powder<br>Nut mix<br>Pistachio cream<br>Pistachio kernels and dried fruits' mix	Barbecue-flavoured snacks mislabelled as chilli-flavoured groundnuts<br>Fruit nut mix<br>Hazelnut paste<br>Hazelnuts<br>Mix containing nuts	Almond butter mislabelled as organic cashew butter<br>Almond paste<br>Bags of pine nuts<br>Baklava<br>Cashew butter

	Confectionery with nuts	Psyllium husk	Nut mix	Aashew nuts incorrectly packaged as student oats (nut and raisin mix)
	Mix of different nuts and corn	Soft almond nougat	Peanut butter	Cashew paste
	Muesli	Spicy groundnuts	Peanut butter mislabelled as organic cashew butter	Peanuts, nuts and mulberries mix
	Salted cashew nuts			Pistachios mislabelled as nougatine with nuts
	Seasoned sunflower seeds			Psyllium husk powder used in frozen bread
	Sesame and sesame paste tahini			Various almond, cashew and peanut butters
	Tahini			
**Other food product / mixed**	Aubergines in oil	Carbonated strawberry and cream flavoured soft drink	Chicken sweet and sour	Canned soybeans mislabelled as spelt
	Cake cream labelled as "lactose free" and "milk protein free"	Carrot and dill hummus	Enzymes	Chilled smoked salmon salads
	Canned peeled tomatoes	Chilled Hawaii chicken salad	Frozen organic mung bean nuggets	Chilled strawberries with chocolate cream
	Chips	Frozen garlic puree	Frozen spring roll sheets	Chilled vegan paprika slices
	Cooked white beans	Frozen potato waffles	Frozen veggie burgers	Croutons used in caesar salad
	Easter egg's paint	Frozen soya product	Melba toast	Frozen beef springrolls
	Gluten-free bread mix	Frozen spicy burgers	Organic coconut sugar	Frozen sandwich
	Gluten-free protein linseed and protein powder	Gluten-free hemp protein	Roasted vegetables	Grilled bell pepper tapenade
	Grilled chicken dish and tuna salad	Gluten-free hummus	Spinach noodles	Olives
	Mini mooncakes	Green olives in glass jars	Various frozen products	Organic gluten-free soy flour
	Organic veggie style chicken chunks mislabelled and packed as veggie style beef strips	Grilled artichokes in sunflower oil	Vegan pate	Organic hummus
	Pickled pepperoni	Instant pumpkin cereal	Vegetarian marinated fillet	Pink roses for cake decoration
	Various foodstuffs	Mini vegetarian burger- nuggets		Spaghetti bolognese
	Various halawa flavours and jams	Olive paste		Spicy peppers filled with tuna and capers

	Vegan dairy-free grated pizza topping	Organic powder preparation for fermented soy dessert		Spinach cream
	Vegan stracciatella flavoured lupin-based yoghurt	Pasta salad with smoked salmon		Spreads
	Wafer sheets	Seitan and vegetable sausages		Various gluten-free processed products
		Soy meat products		Vegetarian burger
		Spicy carrot spread with ginger		Vegetarian sausages
		Sugar free liquorice sweets		
		Vegetable snack		
		Vegetarian mince mistakenly packaged as roasted cubes		
		Vegetarian salami		
**Poultry meat and poultry meat products**	Chilled chicken garlic kiev	Shicken nuggets	Chicken wraps	Chicken pies, duck pies and shredded chicken
	Chilled chicken meatballs with vegetables	Frozen chicken burgers	Chilled canned turkey luncheon meat	Chicken wings
	Chilled vacuum-packed barbecue chicken flatties	Steamed and smoked chicken	Chilled chicken nuggets	Chilled chicken burgers
	Frozen chicken steaks		Frozen breaded chicken chunks	Chilled chicken cubes
	Frozen poultry (chicken and turkey) döner kebab		Frozen chicken burgers and nuggets	Frozen chicken wings
	Sliced chicken cold cuts		Frozen chicken satay	Frozen gluten free chicken nuggets
			Frozen halal battered chicken products	
**Prepared dishes and snacks**	Beef flavour instant rice noodles	Canned Piedmontese tuna salad	Baby food	Canned bean soup
	Bugles chips	Carrot salad	Cheese-flavoured nacho chips mislabelled as plain nacho chips	Chilled Russian salads
	Chilled gluten-free scotch eggs	Chilled bami goreng	Chilled celeriac puree labelled as mashed potatoes	Chips
	Fish and vegetables pie suitable for lactose intolerance	Chilled cheese-flavoured gnocchi	Chilled cottage pie and champ	Crisps
	Frozen apricot dumplings	Chilled chicken Chinese takeaway products	Chilled lamb curry mislabelled as red Thai chicken curry	Curry chicken sandwich mix

Frozen cheese croquettes	Chilled tandoori chicken salad dish	Chilled spicy chicken salad with shrimps mislabelled as chicken with parmesan and red pesto salad	Frozen cheese croquettes
Frozen mashed potatoes mistakenly mixed with potato croquettes	Chinese vegetable spring rolls	Chilled veal with tuna sauce	Frozen seaweed salad
Frozen veal croquettes	crisps	Club salad pasta, tuna and chicken	Frozen tiramisu
Frozen vegan broccoli dumplings	Frozen chicken tikka masala	Frozen beef balls labelled as cheese balls	Frozen vegetarian and vegan snacks
Fruit and nut bar	Frozen vegetarian nuggets	Frozen cooked beef and ale pies	Hummus and aoili
Gluten-free chips	Grill chips	Frozen curry chicken meal incorrectly labelled as sweet and sour chicken meal	Noodles
Gluten-free corn chips	Kashk bademjam	Frozen lamb and carrot dumplings	Peanut snacks
Gluten-free vegetarian nuggets	Ketchup-flavoured chips	Frozen meatballs	Pizza-flavoured pretzels
Instant noodles	Lasagne	Frozen vegan products	Quinoa and kale corn puffs
Italian penne bolognese bake	Makdous	Hazelnut and chocolate rice cake bars	Rice and pea chips
Organic chocolate-filled cookies	Mushroom and bok choy dumplings	Hummus and aioli	Rice salad
Organic lasagne	Noodles with beef and soy sauce incorrectly packaged as noodles with shrimps and curry	Nachos cheese snacks	Sour cream and onion crisps mislabelled as grill crisps
Organic mini apricot bar	Nugget snacks with sesame and seaweed flavor	Noodles	Vegetarian balls with mashed potatoes
Oven baked paprika crisps	Packed snacks	Potato chips	Vegetarian burrito
Pasta filled with goat cheese, honey and walnuts	Pasta salad with peppers and fennel incorrectly labelled as potato salad with spring onion and french cream		
Prepared dish	Pizza	salsa flavoured potato chips	
Prepared sandwiches	Prepared dish	sausage rolls	
Ravioli in minced meat sauce	Prepared vegetables dishes	smoked cod, salmon and king prawn fish pie	
Ready to eat pasta salad with chicken meat	Rendang dish	spring rolls	
Red bean cakes with melon seeds	Salmon lasagne incorrectly labelled as normal lasagne	tangy cheese tortilla chips	

	Roasted potatoes with bacon	Steamed bread filled with chicken	Various snacks	
	salmon wrap	Teriyaki houmous chips	Vegan proteinbars	
	Salt and vinegar flavoured crisps packaged as salted chips	Tortilla wrap		
	Strawberry and banana maize puffs	Waitrose sour cream and chive mix		
	Tortilla chips			
	Vegan snacks			
	Vegan sushibox			
**Soups, broths, sauces and condiments**	Arrabbiata pesto sauce	Andalouse sauce	Apricot and coriander sauce	Broth powders
	Beer mustard	Barbecue sauces	Cheese sauce labelled as Mexican sauce	Caramel topping
	Bottles containing soya sauce	Beef & tomato instant noodles	Curry sauce	Curry paste
	Brandy vinegar 10%	Bolognaise sauce	Curry sauce mix	Pesto
	Chipotle aioli sauce	Canned Chinese chicken soup mislabelled as spicy Chinese soup	Hummus	Red chilli sauce
	Curry tandoori sauce	Carrot and coriander soup	Instant sauce powder for sour roast	Sauce
	Gluten-free cream preparation with potatoes and leeks	Chilled soups	Ketchup	Sichuan sauce
	Honey and mustard dressing	Mirza ghasemi	Mango pickle in oil and garlic pickle in oil	Soups
	Lemon juice concentrate	Pepper sauce	Mayonnaise	Truffle mayo
	Plastic jars of organic beetroot soup containing pumpkin soup	Pesto products	Mushroom & gin sauce	Various syrups and sauces
	Red pesto	Pickles sauce	Organic horseradish	
	Satay sauce	Porcini mushrooms granular stock and bouillon	Pesto	
	Satay sauce mix	Soybean paste with seasoning	Sesame and soy wok sauce mislabelled as teriyaki wok sauce	
	Sauce with clams	Teriyaki sauce	Sriracha chilli sauce	
	Smoked haddock and salmon chowder	Various soups and broths	Truffle sauce	
	Sour mustard	Vegetable soup		
		Wok sauce		

**Wine**	White wine	Rosé wine	Sangria
